# Dislocation of the Os Humeri, upon the Dorsum Scapulæ

**Published:** 1853-05

**Authors:** 


*Dislocation of the Os Humeri, upon the Dorsum Scapulæ, reduced after the expiration of five weeks.* By Paul F. Eve, M. D., Prof. of Surgery in the Nashville University.

Mrs. A. was thrown from a carriage while the horses were running away with it, and in the fall was struck by a wheel upon the left shoulder. This occurred just five weeks, lacking a day, before the dislocation, (the result of this accident,) was reduced. Owing to the great tumefaction which immediately ensued, the peculiar nature of the injury was not detected. When this had subsided, her physicians recognized a dislocation, which was so unusual that she was advised to visit Nashville. Drs. Kelly and Porter examined the case with me on the 21st of November, and we confirmed the opinion already expressed by our professional brethren who had seen it, that there was a dislocation backward of the humerus at the left shoulder joint. This was further strengthened the next day by Drs. Jennings and D. W. Yandell concurring with us.

The symptoms present were a loss of contour in the articulation affected, motion backward and upward of the left arm; flatness of the shoulder, great projection of the coracoid process, prominence of the acromion, hollow under it; a distinct tumor on the dorsum scapulæ behind, and a little below the glenoid cavity; the spinus process of this bone was obscured; the tumor on its dorsum was much nearer its posterior edge than was the head of the humerus on the sound side to the corresponding point of that side; the longitudinal axis of the os humeri was directed behind the glenoid cavity; the left fore-arm was pronated. The inferior extremity of the dislocated limb was longer than the one on the other side. There was no tumor in the axilla, and the elbow of the affected side could be made to approach the chest.

The patient did not now suffer much, but could only use the fore-arm to a limited extent, and the function of the arm was nearly lost.

The peculiar symptoms in the case were the altered direction of the long axis of the arm, the impossibility to carry the elbow backward, the projection of the coracoid process, and the head of the os humeri on the dorsum scapulæ.

Kindly assisted by the gentlemen above mentioned, while one maintained counter-extension by means of a folded sheet in the axilla, (the patient being seated in a chair,) two others extended the limb horizontally outward and forward, with directions to carry it suddenly backward, the head of the os humeri was pressed toward the glenoid cavity, when the reduction was easily effected, without resorting to chloroform or the pulleys. Upon the second trial, probably in three minutes, the bone slipped into its socket with distinct recognition to all present. In a week the patient returned home a distance of about thirty miles.

That the backward dislocation at the shoulder joint is a very rare one, a mere glance into the records of surgery will satisfactorily prove. Its *bibliography* does not extend beyond the present century.

Cases, no doubt, have occurred earlier than this period, but nearly all available in the profession have been derived from modern Surgeons.

In Prof. Pirrie's Principles and Practice of Surgery, 1852, on dislocations *backward*, at the shoulder-joint, he says that "of the head of the humerus on the dorsum of the scapulæ is so rare an accident that Desault had never seen an instance of it; Baron Boyer met with it once in the living body; only two cases occurred at Guy's Hospital in thirty-eight years; in the same number of years Sir Astley Cooper met with two cases, and not more than four cases occurred during his whole professional career; and Mr. Lawrence, in his lectures delivered at St. Bartholomew's Hospital, in 1830, states that at that time he had never seen the humerus dislocated *backward.* After alluding to three or four other cases, and two examples he had met with, he concludes the paragraph by stating that there are on record a few others.

Mr. Bransby Cooper, in his lectures on the Principles and Practice of Surgery, published last year, writes that Boyer, speaking of this accident, says, "there is no well attested instance of dislocation of the humerus outward and backward." He states, however, that he himself had seen several cases, alluding clearly to some of his uncle's Astley; and reminding an American of a similar connecting of E. Holme to the celebrated John Hunter. But he, too, referring to his illustrious relative, remarks, that it was singular that two instances of so rare an accident should occur so closely together in the practice of one individual. In Sir Astley Cooper's great work on dislocations, we find these very cases detailed. In the other Cooper's writings, (Samuel,) he states distinctly a few cases have been recorded. Ferguson has seen one instance; Liston,* Miller and Skey mention none.

During the visit of my colleague, Dr. Buchanan, last year, to St. Bartholomew's Hospital, the first case of dislocation of the head of the humerus on the dorsum of the scapulæ was brought into that institution. Mr. Stanley said it was the first of the kind he had ever seen, and he had been connected with it thirty years. Mr. Lawrence stated that he had met with but one other case in fifty years practice.

In our own country, Dr. Physick, if we recollect right, met with two such dislocations. One was produced by the patient falling into a hatchway and striking the arm near the shoulder-joint upon its edge as the body descended into it. In this instance, the blow or force causing the luxation was applied directly opposite to that which resulted in a similar case here recorded. In my example, the wheel struck the scapulæ posteriorly, carrying it suddenly and forcibly forward, while the arm, fore-arm and hand having no such movement communicated to them, by their dead weight, overcame the slight comparative resistance of the atmosphere, ruptured the scapulo-humeral articulation, and were thrown backward.

In 1831, Dr. George Snyder, of Jackson, Tenn., communicated a case of backward dislocation at the shoulder-joint, to Prof. Gibson, of Philadelphia, in which not succeeding in effecting its reduction as recommended by Sir Astley Cooper, he afterward replaced it by the ordinary means applied to luxation of the os humeri in the axilla. Dr. S. made the very sensible remark, that producing a secondary, or consecutive displacement of the humerus downward, which some authors recommend, cannot facilitate the reduction. To be reduced from its second position, it must necessarily increase the rupture in the ligaments or soft parts, or describe a curve to enter again the glenoid cavity.

The case now recorded we believe is the first of the kind occurring in, or about Nashville.—*N. Y. Med. Gazette.*

---

* See Elements.

